# Nano-Enabled Potentiation of a Lead Mono-Carbonyl Curcumin Analogue via PEGylated Graphene Oxide for Enhanced Glycemic Control

**DOI:** 10.3390/pharmaceutics18050568

**Published:** 2026-05-02

**Authors:** Babar Ayub, Haya Hussain, Farman Ali Khan, Nasir Mehmood Khan, Abid Ullah, Kifayat Ullah, Syed Wadood Ali Shah, Jian Wang, Shujaat Ahmad

**Affiliations:** 1 Department of Pharmacy, Shaheed Benazir Bhutto University Sheringal Dir (Upper), Khyber Pakhtunkhwa 18000, Pakistan; babarayub18@gmail.com (B.A.); haya@sbbu.edu.pk (H.H.); abid@sbbu.edu.pk (A.U.); ullahkifayat0@gmail.com (K.U.); 2 Department of Chemistry, Shaheed Benazir Bhutto University Sheringal Dir (Upper), Khyber Pakhtunkhwa 18000, Pakistan; farmanali@sbbu.edu.pk; 3 Department of Agriculture, Shaheed Benazir Bhutto University Sheringal Dir (Upper), Khyber Pakhtunkhwa 18000, Pakistan; nasir@sbbu.edu.pk; 4 Department of Pharmacy, University of Malakand, Chakdara, Dir (Lower), Khyber Pakhtunkhwa 18300, Pakistan; pharmacistsyed@gmail.com; 5 School of Chinese Materia Medica, Chongqing University of Chinese Medicine, Chongqing 402760, China; wangjian@cqctcm.edu.cn; 6 College of Traditional Chinese Medicine, Chongqing Medical University, Chongqing 400016, China

**Keywords:** type 2 diabetes, mono-carbonyl curcumin analogues, molecular docking, hyperglycemia, lipid profile, PEGylated graphene oxide, nanoformulation

## Abstract

**Background:** The global healthcare system faces a significant challenge due to the escalating prevalence of type 2 diabetes, affecting over 10% of the world’s population. Suppression of postprandial hyperglycemia through inhibition of carbohydrate-hydrolyzing enzymes is an effective therapeutic strategy. Although curcumin effectively inhibits α-amylase and α-glucosidase activities, its lower solubility and bioavailability restrict its clinical application. In this study, five mono-carbonyl curcumin analogues (CA1–CA5) were synthesized and evaluated for their antidiabetic potential following selective experimental methods both in vitro, and in vivo. Enhanced delivery for the most potent analogue was achieved through PEGylated graphene oxide (PEG-GO) to overcome the shortcomings of curcumin compounds. **Methods:** In silico ADME profiling was conducted using SwissADME, and molecular docking studies were performed with AutoDock Vina (v1.5.7) to assess enzyme binding interaction. The synthesized compounds were further evaluated using in vitro α-amylase and α-glucosidase inhibition assays, followed by in vivo blood profile analysis. The most active analogue CA3 (chloro derivative) was loaded onto PEG-GO and characterized using UV–visible spectroscopy, Fourier-transform infrared spectroscopy, and scanning electron microscopy. **Results:** Among all of the compounds, CA3 exhibits the strongest binding affinity and highest enzyme inhibitory activity, followed by CA2 and CA4. PEG-GO-CA3 demonstrated significantly enhanced biological activity compared to its free form. In vivo studies showed marked improvements in body weight and lipid profile, along with significant reductions in blood glucose, glycated hemoglobin, urea, creatinine, alanine aminotransferase, and aspartate aminotransferase levels over a 28-day treatment period as compared to a diabetic control. Spectroscopic and morphological analyses confirmed successful loading of CA3 onto PEG-GO (27.7–31.5%) with a release profile of 38–57% after 12 and 36 h in a controlled environment at pH 7. **Conclusions:** These findings suggest that PEG-GO-loaded mono-carbonyl curcumin analogues represent promising therapeutic candidates for the management of T2DM.

## 1. Introduction

Diabetes mellitus (DM) is a complex metabolic disorder characterized by dysregulation in the metabolism of carbohydrates, lipids, and proteins, mainly due to insufficient insulin secretion or resistance to its action, resulting in sustained hyperglycemia [1]. As the most rapidly increasing endocrine and metabolic disease globally, the pathophysiology of diabetes is primarily associated with impaired insulin dynamics, either inadequate production, insulin dysfunctional signaling, or both [2]. Type 2 diabetes mellitus (T2DM), the predominant form, is marked by chronic hyperglycemia and disrupted carbohydrate metabolism, and is recognized as a major contributor to global morbidity, mortality, and economic strain [3]. According to the International Diabetes Federation (IDF), the global prevalence of T2DM reached 536.6 million adults (10.5%) in 2021, and it is expected to rise to 783.2 million (12.2%) by 2045 [4]. All compounds demonstrate predicted high gastrointestinal absorption, rapid urbanization, economic development, sedentary behavior, dietary changes, and population aging, particularly in low- and middle-income countries. Currently, over 90% of all diabetes cases are classified as T2DM [5].

T2DM arises from defects in insulin production and/or its biological activity, leading to chronic hyperglycemia that progressively damages multiple organ systems, particularly the vascular system [6]. The associated complications of diabetes include diabetic retinopathy, nephropathy, neuropathy, and angiopathy. One therapeutic approach for managing non-insulin-dependent diabetes mellitus (NIDDM) involves mitigating postprandial hyperglycemia by limiting intestinal glucose absorption. This is achieved by inhibiting key enzymes responsible for the hydrolysis of complex carbohydrates into absorbable monosaccharides [7]. Among these, α-glucosidase plays a pivotal role. It is an intestinal, membrane-bound enzyme widely present in animals, plants, and microorganisms, catalyzing the cleavage of α-glucose units from the non-reducing ends of oligosaccharides and disaccharides [8,9]. Located in the brush-border epithelium of the small intestine, α-glucosidase facilitates the terminal digestion of carbohydrates into glucose, which is subsequently absorbed into the bloodstream. Pharmacological inhibition of this enzyme delays carbohydrate digestion, thereby attenuating postprandial glucose excursions and improving glycemic control in T2DM patients [10].

Pancreatic α-amylase is a calcium-based metalloenzyme responsible for hydrolysis of α-1,4 glycosidic bonds of polysaccharides such as amylopectin, amylase and glycogen. The final phase of carbohydrate digestion is complete by α-glucosidase which acts on 1,4-alpha bonds to generate glucose [11]. Numerous plant-derived phytochemicals and synthetic agents have demonstrated the ability to inhibit α-amylase activity, thereby contributing to the attenuation of postprandial blood glucose levels [12].

Curcumin is a naturally occurring polyphenol found in *Curcuma longa*, family Zingiberaceae, commonly called turmeric and has potential uses in diabetes, inflammation, cancer, infections, neurodegenerative disorder, and oxidative stress. Traditional Chinese medical practitioners use curcumin in the management of diabetes [13,14,15]. Curcumin improves insulin resistance and decreases glucose, insulin and leptin levels. It reduces inflammation by inhibiting tumor necrosis factor α and interleukin IL6-1β in patients suffering from T2DM [16]. The US Food and Drug Administration (FDA) has classified curcuminoids as generally safe. Clinical trials have shown that they are safe at doses up to 8000 mg/day. These curcuminoids exhibit excellent α-glucosidase and α-amylase inhibitory potential, likely due to strong van der Waals interactions with the amino acid residues of both enzymes. These compounds also decrease postprandial hyperglycemia, improve lipid profile, reduce glycosylated hemoglobin (HbA1c), and exhibit liver- and kidney-protective functions. Furthermore, they have been used in the management of hyperglycemia and type 2 diabetes mellitus (T2DM) [17]. Many curcumin analogues and derivatives have been shown to possess antidiabetic potential by reducing oxidative stress and inflammation and modulating the pathways responsible for hyperglycemia [18,19]. Curcumin and its analogues effect many molecular targets responsible for diabetes. It has the potential to inhibit α-amylase and α-glucosidase activities; reducing postprandial hyperglycemia by reducing intestinal glucose absorption. By inhibiting pancreatic amylase, these curcumin analogues reduce intestinal side effects [20].

PEGylated graphene oxide (PEG-GO) has emerged as an advanced nanocarrier due to its unique physicochemical properties and improved biocompatibility [21]. The incorporation of polyethylene glycol onto the graphene oxide surface enhances its water dispersibility, reduces aggregation, and minimizes potential cytotoxicity, therefore making it suitable for biomedical applications. Its high surface area and abundance of functional groups provide multiple binding sites for efficient drug attachment, while PEG chains prolong the circulation time and improve systemic stability. Loading curcumin or its analogues onto PEG-GO improves the inherent limitations of curcumin such as poor solubility and rapid metabolism. This also ensures sustained release, improved cellular uptake, and enhanced therapeutic efficacy [22]. In this study, we explored the antidiabetic potential of five synthesized curcumin analogues. Their efficacy was evaluated through in vitro enzyme inhibition assays, in vivo animal experiments, and molecular docking studies, providing valuable mechanistic insights into their therapeutic action. This was followed by the synthesis of PEGylated GO, onto which the most potent curcumin analogue was loaded to ensure its enhanced aqueous solubility and improve bioavailability.

The high attrition rate in drug development has driven the use of in silico methods to predict pharmacokinetic and toxicity profiles, offering faster and more cost-effective alternatives to experimental studies. Computer-aided drug design is widely applied for hit identification, lead optimization, and molecular interaction analysis. Tools such as SwissADME enable rapid evaluation of key properties, including physicochemical characteristics, solubility, pharmacokinetics, and drug-likeness, making them valuable in early-stage drug discovery [23]. There has been no previous work reported on the computational studies of these synthesized curcumin analogues against α-amylase/glucosidase as well as in vitro and in vivo testing in diabetic models. Hence, the aim of this study was to synthesize and evaluate mono-carbonyl curcumin analogues (CA1–CA5) for their antidiabetic potential using in silico, in vitro and in vivo experiments. Moreover, the most potent analogue was loaded onto PEGylated graphene oxide to evaluate for its enhanced bioavailability, sustainable drug delivery and effectiveness in diabetes management.

## 2. Materials and Methods

### 2.1. Material

All of the chemicals such as acarbose (Ac), quercetin, streptozocin (STZ), α-glucosidase and α-amylase, graphene oxide (GO), polyethylene glycol (PEG), Trichloroacetic acid (TCA) and sodium hydroxide (NaOH) were obtained from Sigma-Aldrich (Hamburg, Germany). All reagents and solvents employed in the study were of analytical-grade quality.

### 2.2. Methods

**Figure 1:**
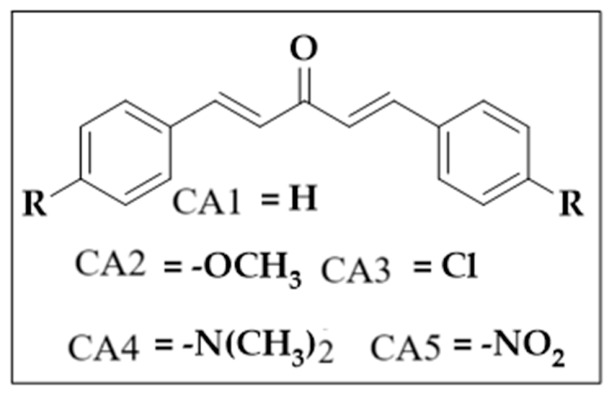
Five synthesized mono-carbonyl curcumin analogues.

The mono-carbonyl curcumin analogues (CA1-CA5) as shown in Figure 1 were already synthesized by our research group [24].

#### 2.2.1. SwissADME Study

SwissADME was employed to preliminarily assess the drug-likeness and oral bioavailability potential of the synthesized compounds. The 2D structures of the synthetic derivatives were drawn via ChemDraw Ultra version 10. The structures of these compounds were imported and the structure SMILES of each compound was entered. A SwissADME [23] drug design study was run to generate the data [25]. The generated data was noted for analysis [26]. SwissADME is freely accessible at http://www.swissadme.ch. (Access date: 30 December 2025)

#### 2.2.2. Molecular Docking

The molecular interactions between the selected ligands and target proteins were examined using AutoDock Vina (version 1.5.7). Prior to docking, the enzyme structures were prepared by removing non-essential ligands and water molecules with BIOVIA Discovery Studio 2021. All ligand structures were subjected to energy minimization using Spartan 14 (version 1.1.4). Polar hydrogens were added to the protein structures in AutoDock Vina, and Kollman charges were assigned. Ligand partial charges were calculated using the Gasteiger method. The active sites of the proteins were identified using the Computed Atlas of Surface Topography of Proteins (CASTp) version 3.0 (http://sts.bioe.uic.edu/castp/index.html?5tsp) (Access date: 30 December 2025). For α-glucosidase (PDB ID: 3A4A), the docking grid center was set at coordinates x: 20.53, y: −8.271, z: 22.38 with grid dimensions of x: 40, y: 40, z: 40. For α-amylase (PDB ID: 1HNY), the grid center was x: 2.728, y: 43.57, z: 17.101 with grid dimensions of x: 60, y: 60, z: 60. The grid spacing was set to 0.375 Å. Finally, interactions and bonding types between the ligands and target proteins were visualized and analyzed using Discovery Studio Visualizer and LigPlot+ (version 2.2.8).

#### 2.2.3. In Vitro Study

##### In Vitro α-Glucosidase Inhibitory Assay

The inhibitory potential of the mono-carbonyl curcumin analogues (CA1-CA5) was determined against α-glucosidase. The test samples contained 10 µL of each (CA1-CA5) at various concentrations, 20 µL of α-glucosidase (0.5 unit/mL), and 120 µL of 0.1 M phosphate buffer (pH 6.9). The solution was incubated in 96-well plates at 37 °C for 15 min. The reaction was started by addition of 20 µL (0.5 mM) of the p-nitrophenyl-D-glucopyranoside. The reaction was stopped by addition of 80 µL (0.2 M) sodium carbonate solution and absorbance was measured at 405 nm. A reaction system without samples served as a positive control to account for background absorption, and a blank without glucose was used. α-Glucosidase was not used in the positive control, and instead a sample-free reaction technique was employed to adjust the background absorbance of the blank [27].

##### In-Vitro α-amylase Inhibitory Assay

The α-amylase inhibitory activity of the mono-carbonyl curcumin analogues (CA1–CA5) was assessed following a standardized protocol. A starch solution was prepared by dissolving 2 g of starch in 80 mL of 0.4 M NaOH and heating the mixture at 80 °C for 30 min. The solution was then cooled using an ice bath, neutralized with 2 M HCl, and its pH adjusted to 6.9 with distilled water. Different concentrations of samples were prepared in a phosphate buffer system. Acarbose was used as a positive control. In total, 5 μL of sample, 35 μL of substrate and 35 μL of phosphate buffer (pH 6.9) were mixed and incubated for 30 min. After this, 20 μL of the enzyme solution (50 μg/mL) was introduced and the mixture was incubated for 30 min. The reaction was terminated by adding 50 μL of 0.1 M HCl, and absorbance was recorded at 580 nm [28].

#### 2.2.4. In Vivo Study

##### Animals

Male Wistar rats aged 10–12 weeks and weighing between 205 and 215 g were procured from the National Institute of Health, Islamabad. The animals were housed under standard laboratory conditions, including a controlled temperature of 25 ± 2°C, relative humidity of 55–65%, and a 12 h light/dark cycle. Water and a standard diet were also offered. Animals were handled according to the institutional ethical committee of Shaheed BB University Dir Upper (SBBU/IEC/25-10).

##### Assessment of Acute Toxicity Study

To evaluate the potential toxic effects and identify a safe and optimal dosage for in vivo studies, acute toxicity assessments were conducted using rats. These tests aimed to establish the safety profile of the compounds CA1 to CA5. Various groups of mice received oral doses of the compounds at 5, 15, 30, 50, 75, 100, and 150 mg/kg of body weight. The animals were carefully observed for any signs of toxicity over a 24 h period, such as convulsions, tremors, changes in motor activity, loss of the righting reflex, lacrimation, diarrhea, muscle spasms, salivation, sedation, or hypnosis. Further observations were continued for up to 72 h to monitor any mortality. No deaths or severe adverse effects were noted up to the highest administered dose of 150 mg/kg. Based on these results and following OECD guidelines, 15 mg/kg equivalent to one-tenth of the maximum tested dose was selected as the suitable dose for subsequent in vivo investigations of the mono-carbonyl curcumin analogues [29].

##### Type II Diabetes Induction

Following adaptation, 50 mg/kg of streptozotocin (STZ) dissolved in 0.1 M citrate buffer was injected intraperitoneally (i.p.) into HFD rats. Rats were administered a 10% glucose solution for three days to lower hypoglycemic-shock-related mortality. At 72 h after STZ therapy, blood glucose levels were measured in a tail vein using an SD glucometer (ACCU-CHECK, Roche, Seoul, Republic of Korea). Fasting blood glucose levels in diabetic animals exceeded 250 mg/dL [30].

##### Experimental Design for Anti-Diabetic Activity

The animals were divided into thirteen groups (*n* = 5) each after induction. These groups consisted of a control group, diabetic group, positive control (acarbose 20 mg/kg) and ten test groups. Five test groups (CA1-CA5) received unloaded CA and five test groups (CA1-CA5) received CA loaded onto PEG-GO. The animals in the control (normal) group and the STZ diabetes groups received only the vehicle. The compounds were given orally (15 mg/kg) to the treated animals for a period of four weeks [31].

##### Calculating Body Weight and Blood Sugar Levels

Measurements of blood sugar and body weight were taken on the first day and every seven days using a digital glucometer [32].

##### Glycated Hemoglobin Level

As per the manufacturer’s guidelines, the levels of glycated haemoglobinA1c (HbA1c) were measured in each therapy group as per standard protocols [33].

##### Assessment of Serum Profile

At the end of the 28-day study period, following the completion of the antidiabetic evaluation, all animals were humanely euthanized using isoflurane anesthesia. Blood samples were then collected to analyze various biochemical parameters. The assessment included measurements of liver enzymes such as aspartate transaminase (AST) and alkaline phosphatase (ALP), as well as lipid profile components including total cholesterol (TC), triglycerides (TGs), high-density lipoprotein (HDL), and low-density lipoprotein (LDL). Kidney function markers, namely urea and creatinine, were also evaluated [34].

#### 2.2.5. Synthesis of PEGylated GO/CA3 Nanocomposite

##### Graphene Oxide Synthesis

GO was synthesized using a modified Hummer’s method. In summary, 1 g of graphite powder was mixed with 50 g of NaCl and ground to powder. After removal of NaCl by water and filtration, the fine graphite powder was dissolved in 30 mL (90%) sulfuric acid and stirred for 10 h. Afterwards, KMnO_4_ (9 g) was added batchwise while keeping the temperature below 10 °C in a water bath. The mixture was then continuously stirred for 40 min at 35–40 °C and for a further 40 min at 70 °C to 80 °C, followed by dropwise addition of 50 mL distilled water (DW) and continuous stirring for 30 min at 90 °C. This was followed by the addition of 10 mL hydrogen peroxide (30%) to terminate the reaction. From the final mixture, GO was suspended in DW for several days and separated by washing with 5% HCl solution through repeated centrifugation. The supernatant was discarded and GO was dried in an oven at 65 °C [35].

##### Carboxylation of GO

GO was activated through carboxylation of its surface groups. In short, GO (50 mg) was homogeneously dispersed in 50 mL DW and was bath-sonicated for 1 h. After this, 6 g, NaOH and 5 g TCA were dissolved in 250 mL of DW and added to the GO suspension (pH: 7) and further sonicated for 3 h, filtered by a 14 kDa dialysis membrane overnight, washed thoroughly with DW and finally dried in the oven [21].

##### Synthesis of PEG-GO Nanocomposite

A modified method proposed by Li et al., 2019 was used for synthesis of PEG-GO; in short, carboxylated GO (200 mg) was dissolved in 50 mL anhydrous ethanol and bath-sonicated for 1 h at room temperature. A solution of PEG (6000 dp 200 mg/100 mL) was added dropwise to the above solution and stirred at 70 °C for 6 h. The obtained suspension was centrifuged at 14,000 rpm to remove unbound PEG. The obtained pellets were washed several times with DW and finally dried in the oven [36].

##### Loading of Curcumin Analogues on PEG-GO

The method of Zhu et al., 2014, was used to load curcumin analogues on PEG-GO. Briefly, 10 mg of PEG-GO was dissolved in DW and stirred for 30 min. A solution containing a curcumin analogue (1 mg/3 mL in ethanol) was added to PEG-GO solution and stirred overnight. The free CA was removed by dialysis membrane in ethanol for 3 days, followed by UV/visible analysis and determination of drug-loading capacity using the following formula [36,37].

$$\mathrm{CA}\;\mathrm{loading}\;\%=\frac{Free\;CA-PFGGOCA}{Free\;CA}\times 100$$

##### Drug-Release Study

Drug release from PEG-GO was measured at pH 2, 6.8, and 7.4, corresponding to stomach, intestinal, and blood pH, respectively. Simulated gastric and simulated intestinal fluids were prepared [38]. In total, 10 g sodium chloride and 0.2 g potassium chloride were added to 100 mL sodium phosphate buffer system (20 mmol, pH 6.7) to maintain pH 7.4 similar to blood pH followed by the addition of a small amount of 2% Tween 40. PEG-GO-CA (5 mg/20 mL) was added to the above solution and incubated at 37 °C. This was followed by transferring the suspension to a beaker containing 14 kDa dialysis membrane placed in 20 mL release medium. After a specific interval of time, a 2 mL aliquot was taken for measuring CA concentration in the release medium through spectroscopy [39].

##### Characterization

A BMS spectrophotometer (VIS-1100, NYC, New York, NY, USA) was use for optical density measurement. The Agilent Cary 660 FT-IR spectrometer was used for FTIR spectral measurement (600–4000 cm^−1^) using ATR mode. The surface morphology was examined using a scanning electron microscope (SEM, TESCAN VEGA, LMU, Brno, Czech Republic). The samples were coated with a layer of gold using a Quorum SC7620 sputter-coater. Images were captured at different magnifications. Dynamic Light Scattering (DLS) measurements were conducted on a BeNano 180 Zeta instrument (Bettersize, Dandong, China) at 25 °C. Samples were dispersed in deionized water or phosphate-buffered saline (PBS, pH 7.4) at a concentration of 0.1 mg/mL and sonicated for 5 min prior to measurement. Zeta size, hydrodynamic diameter and polydispersity index (PDI) were determined from three independent measurements.

#### 2.2.6. Statistical Analysis

All results were expressed as the mean ± standard deviation (SD), with a sample size of three (*n* = 3). Statistical analysis was performed using one-way ANOVA followed by Dunnett’s post hoc test for multiple comparisons in GraphPad Prism version 5.01 to determine the significance of the observed differences.

## 3. Results

### 3.1. Pharmacokinetic Profile

All five derivatives (CA1–CA5) satisfied Lipinski’s rule of five and other drug-likeness criteria, indicating favorable oral drug-like properties. Their physicochemical parameters, including molecular weight (234.2–324.2 g/mol), hydrogen bond donors and acceptors, topological polar surface area, rotatable bonds (4–6), and molar refractivity (75.9–104.3), remained within acceptable ranges. SwissADME analysis showed variable lipophilicity, with CA3 being more lipophilic and CA5 moderately lipophilic, while most derivatives exhibited moderate aqueous solubility. All compounds demonstrate predicted high gastrointestinal absorption and blood–brain barrier permeability, with CA4 being the only P-glycoprotein substrate. CYP inhibition profiling revealed consistent inhibition of CYP2C19 and CYP2C9 across all derivatives, while selective inhibition patterns were observed for CYP1A2, CYP2D6, and CYP3A4. All compounds displayed a uniform bioavailability score of 0.55, complied with major drug-likeness filters, showed no PAINS alerts except CA5, and exhibited limited Brenk structural alerts.

### 3.2. Molecular Docking Studies of Compounds Against a-Amylase

**Figure 2:**
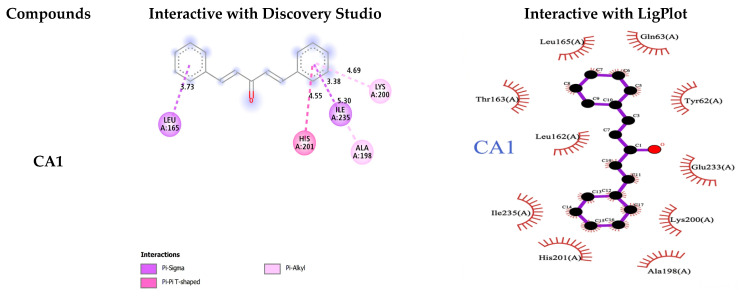

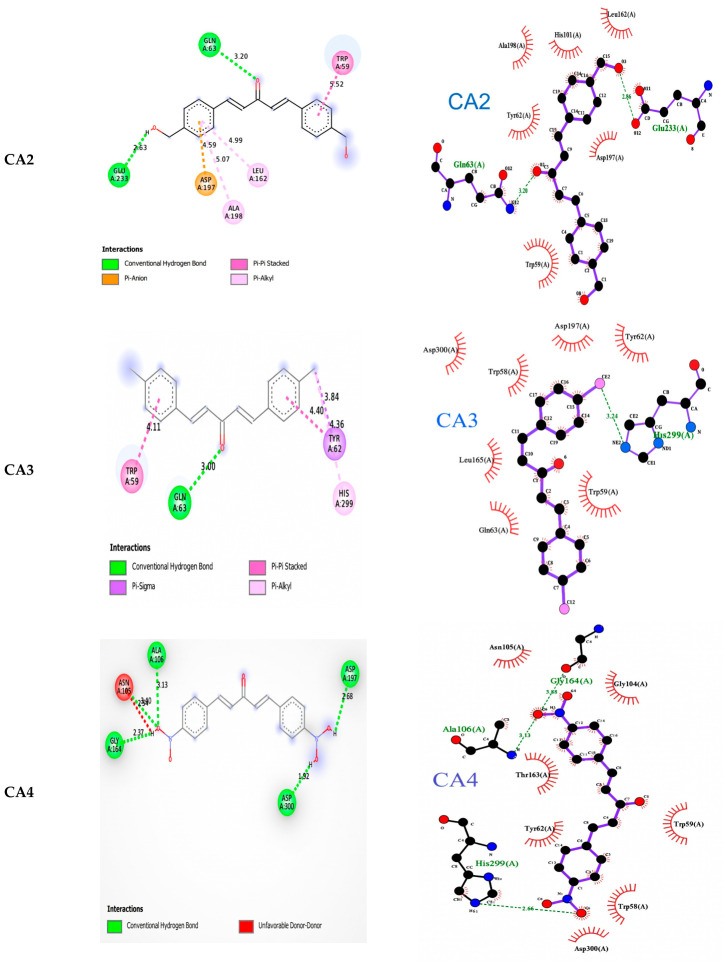

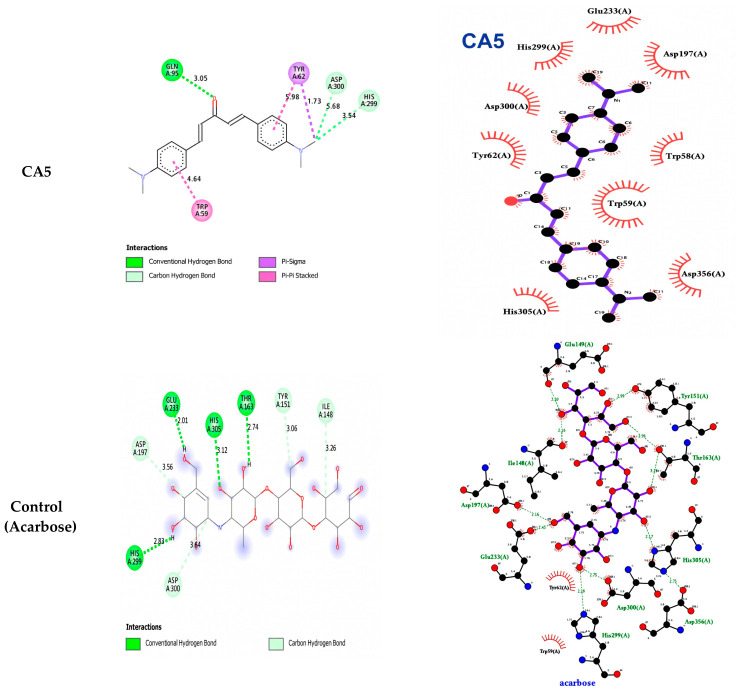
Interactions of ligands with α-amylase proteins.

In the docking study of the compounds with a-amylase, all compounds showed excellent binding affinity with α-amylase as shown in Table 1 and Figure 2. CA3 exhibited the highest binding affinity, with a binding energy of−10.1 ± 0.3 kcal/mol, indicating strong interaction with α-amylase. CA3 formed a strong hydrogen bond with Gln63 (3.00 Å), along with hydrophobic and other bond interactions involvingTrp59 (4.11 Å), His299 (4.36 Å), and Tyr62 (3.84 and 4.40 Å) of α-amylase. This binding strength is followed by CA2 and CA4. The binding affinity of the compounds decreased in the order CA3 > CA2 > CA4 > CA5 > CA1.

### 3.3. Molecular Docking Studies of Compounds Against α-Glucosidase

**Figure 3:**
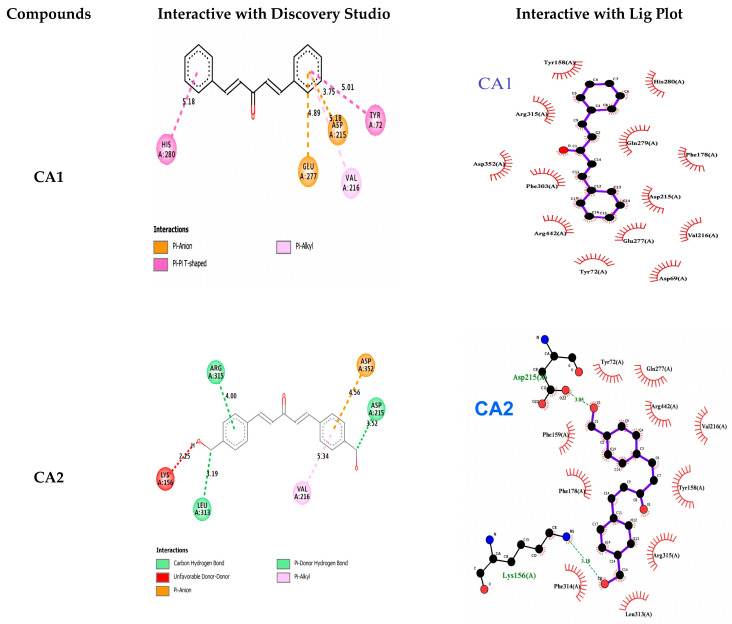

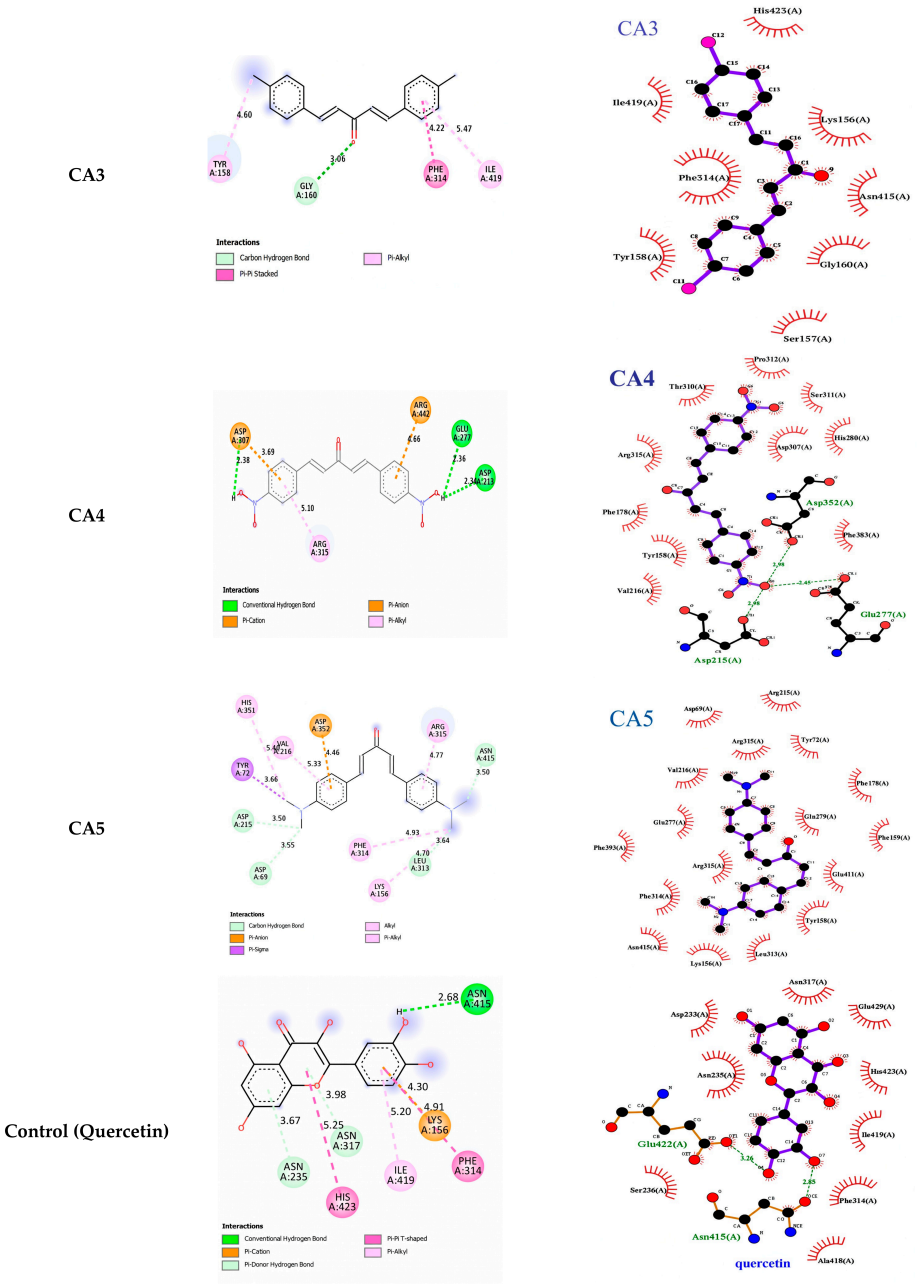
Interactions of ligands with α-glucosidase proteins.

In the docking studies with α-glucosidase, all of the ligands showed promising binding affinities, especially CA3, CA2 and CA4, which showed a better affinity of −10 ± 0.32 kcal/mol, −9.8 ± 0.6 kcal/mol and −9.3 ± 0.65 kcal/mol as compared to quercetin, which was used as a positive control having a binding energy of −9.9 ± 0.3 kcal/mol. CA3 shows strong hydrogen bond interactions of the amino acid Gly160 (3.06 Å) and hydrogen and other hydrophobic bond interactions of the amino acids Tyr158 (4.60 Å) Phe314 (4.22 Å) Ile419 (5.47 Å) of α-glucosidase. This is followed by CA2 and CA4. The order of binding affinity of these curcumin analogues from high to low is CA3, CA2, CA4, CA5 and CA1 (Table 2 and Figure 3).

### 3.4. In Vitro α-Glucosidase and α-Amylase Inhibition Study

Table 3 shows CA3 is highly potent against the tested enzymes with obtained IC_50_ values of 24.1 ± 2.7 µM against α-glucosidase and 18.4 ± 1.8 µM against α-amylase, respectively, as compared to acarbose at 21.4 ± 1.6 µM and quercetin at 15.5 ± 1.7 µM. It is followed by CA2 and CA4. The order of compounds with potency from high to low is CA3, CA2, CA4, CA5, and CA1. The chloro derivatives are highly potent due to the inductive effect caused by chlorine atoms that tend to make the compounds more reactive as compared to others. CA3 loaded onto PEG-GO shows higher potency as compared to unloaded CA3 with IC_50_ values of 20.2 ± 0.95 and 16.1 ± 1.1 µM against α-amylase and α-glucosidase, respectively.

### 3.5. In Vivo Studies 

#### 3.5.1. Effect of Curcumin Analogues on Blood Glucose Level

Table 4 shows the blood glucose concentration of the diabetic group, positive control group, normal control group, and experimental group over 28 days. The blood glucose level of all groups was analyzed every seven days. Among the six mono-curcumin analogues, CA3 showed the highest activity. At day 1, it significantly decreased the glucose concentration from 377 ± 3 mg/dL to 310 ± 3 mg/dL as compared to diabetic control which was 350 ± 4. It is followed by CA2 and CA4. Over twenty-eight days, acarbose, CA3, CA2 and CA4 decreased the glucose concentration to 145 ± 3 mg/dL, 234 ± 2 mg/dL, 256 ± 3 mg/dL and 295 ± 1 mg/dL, respectively, as compared to the diabetic group at 384 ± 3 mg/dL. CA3 loaded onto PEG-GO showed higher activity as compared to unloaded CA3. This nanoformulation reduced the blood glucose concentration to 191 ± 2 mg/dL by the 28th day, whereas free CA3 only decreased it to 234 ± 2 mg/dL. The activity of loaded CA3 was comparable to that of acarbose (145 ± 3 mg/dL), indicating that loading CA3 onto PEG-GO enhances its potency.

#### 3.5.2. Effect of Curcumin Analogues on Lipid Profile of Animals 

Table 5 presents the lipid profiles of all experimental groups. All tested compounds showed an improvement in the lipid profiles of the treated animals. Among them, CA3 exhibited the most significant biological activity compared to the diabetic control group, followed by CA2 and CA4. CA3 reduced triglyceride (TG) levels from 138 ± 2 mg/dL to 106 ± 2 mg/dL, total cholesterol (TC) from 74 ± 1 mg/dL to 61 ± 2 mg/dL, and low-density lipoprotein (LDL) from 23 ± 1 mg/dL to 17 ± 1 mg/dL vs. their respective diabetic control. Additionally, CA3 improved high-density lipoprotein (HDL) levels, from 17 ± 1 mg/dL to 23 ± 0.2 mg/dL, indicating a favorable shift in lipid metabolism relative to the diabetic control. CA3 loaded onto PEG-GO showed higher activities as compared to unloaded CAs.

#### 3.5.3. Effect of Curcumin Analogues on Glycosylated Hb (Hb %) of Animals

Table 6 shows the levels of glycosylated hemoglobin (HbA1c, %) in all experimental groups. Treatment with CA3 resulted in a reduction in HbA1c levels to 6.3 ± 0.2, this is followed by CA2 (7.1 ± 0.3) and CA4 (7.7 ± 0.4) as compared with the diabetic control group (10.3 ± 0.5). CA3 loaded onto PEG-GO demonstrated superior activity compared to the corresponding unloaded curcumin analogues, indicating enhanced therapeutic efficacy upon nanoformulation.

#### 3.5.4. Effect of Curcumin Analogues on Urea, Creatinine, ALT and AST Level of Animals

Table 7 shows the urea, creatinine, ALT and AST levels in all groups under observation. CA3 significantly decreased the urea level from 103 ± 3 mg/dL to 56 ± 3 mg/dL, creatinine from 2.1 ± 0.03 mg/dL to 1.5 ± 0.04 mg/dL, ALT from 126.4 ± 3 U/L to 86 ± 1 U/Land AST from 103.4 ± 2 U/L to 76 ± 2 U/L as compared to the diabetic group; it is followed by CA2 and CA4. CA3 loaded onto PEG-GO shows higher activities as compared to unloaded CAs.

#### 3.5.5. Effect of Curcumin Analogues on Body Weight of Animals

Table 8 shows the weight changes of all groups under observation over 28 days. All compounds significantly improved the body weight of the animals. CA3 showed the greatest activity, followed by CA2 and CA4. Treatment with CA3 restored animal weight to 192 ± 3 g, compared to 189 ± 4 g for CA2, 191 ± 4 g for CA4, and 171 ± 2 g for the diabetic control group. CA3 loaded onto PEG-GO–loaded showed higher potency than unloaded CA analogues.

### 3.6. PEGylated GO/CA3 Nano-Composite Analysis

#### 3.6.1. UV/Visible Analysis

**Figure 4:**
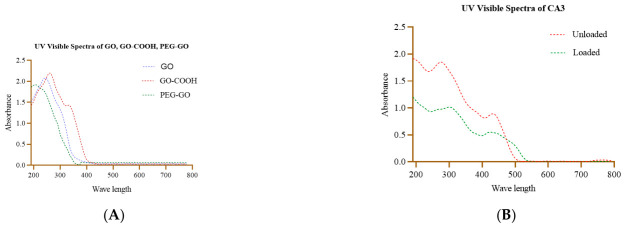
(**A**). UV/visible spectra of GO, GO-COOH and PEG-GO (**B**). UV/visible spectra of CA3 unloaded and CA3 loaded onto PEG-GO.

UV/visible spectra were obtained for GO, GO-COOH, PEG-GO, unloaded CA3 and CA3 loaded onto PEG-GO as shown in Figure 4A,B. GO provided a characteristic peak at 240 nm and 310 nm due to π-π * (C=C), n-π * (C=O). A red shift to 265 nm and 335 nm was noted due to the carboxylation of GO. These signals decreased (blue shift) due to PEGylation of GO because PEG masked signals of the electronic transitions in PEG-GO. These changes in signals and intensities indicated carboxylation and successful PEGylation of GO (Figure 4A). CA3 gave its characteristic absorption pattern. A red shift and decrease in intensities have been noted in its spectra when loaded onto PEG-GO. These spectral shifts confirmed the successful loading of CA3 onto PEG-GO (Figure 4B).

#### 3.6.2. FTIR Spectral Analysis

##### FT-IR Analysis Spectra of GO-COOH

**Figure 5:**
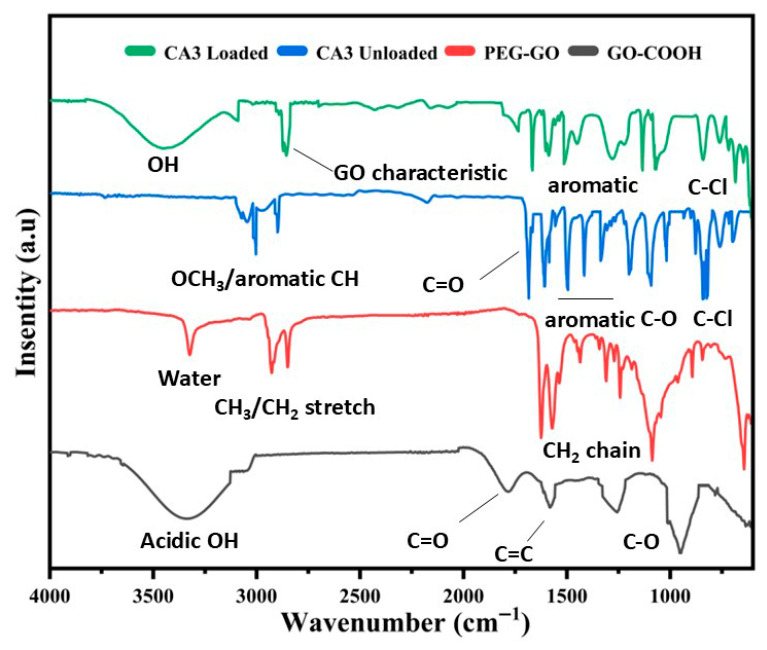
FTIR spectra of GO-COOH, PEG-GO, CA3 unloaded and CA3 loaded onto PEG-GO.

The FTIR spectrum of carboxylated graphene oxide (GO-COOH) indicated the successful introduction of carboxyl groups onto the graphene oxide framework as shown in Figure 5. The observed absorption peaks correspond to various oxygen-based functional groups attached to the graphene surface. A broad and intense band around 3400 cm^−1^ is due to the O-H stretching vibration of the hydroxyl group of carboxylic acid groups; the peak appearing near 1720 cm^−1^ is due to the C=O stretching vibration from carbonyl groups of carboxylic acid. The absorption band around 1620 cm^−1^ is assigned to the C=C skeletal vibration, while the bands in the region of 1050–1250 cm^−1^ represent the C-O stretching vibrations of epoxy and alkoxy groups in the graphene sheet (Figure 5).

##### FT-IR Analysis of PEG-GO, Free and Nanoformulated CA3

As shown in Figure 5, in the FT-IR spectrum of PEG-GO, after the PEGylation of GO, distinct changes were observed. It showed absorption at 3400 cm^−1^ due to the O-H stretching vibration of water. The reduction in broadness of OH stretching is due to the formation of ester bonds as a consequence of PEG binding to the COOH terminal groups. The strong absorption at 2890–2920 cm^−1^ and 1400 cm^−1^ is due to CH_2_ stretching and bending vibration which is a characteristic feature of the methylene chain. The reduction in absorption at 3300 cm^−1^ is due to reduced acidic and slightly enhanced OH group functionalities. A sharp and strong peak at 1100 cm^−1^ indicates the presence of C-O-C ether linkage from the PEG chain. The decrease in intensity of carboxyl (C=O) and carboxylate groups (COO-) around 1700 cm^−1^ and 1620–1640 cm^−1^ indicates covalent functionalization of GO through ester linkage with PEG.

CA3 gave a characteristic FTIR spectrum. A strong band around 1660–1670 cm^−1^ showed the presence of a carbonyl C=O stretch. The band around 1570 cm^−1^ indicated the CA3 framework. A stretch at 1625–1600 cm^−1^ indicated the presence of an aromatic C=C bond. Aromatic bands at 1590–1500 cm^−1^ and an out-of-plane C–H band at 760–700 cm^−1^ showed phenyl rings. An aryl–Cl band at 1090–1030 cm^−1^ and its out-of-plane (OOP) band at 840–830 cm^−1^ indicated a chlorophenyl group. The FTIR spectrum of PEG-GO-CA3 shows broadening of the O-H band (3400), and red shift in the C=O band (1650–1660 cm^−1^) which indicate hydrogen-bonding and π–π interactions with the GO basal plane. Other signals show that the linkages amongst CA3, PEG and GO are intact (Figure 5).

##### SEM Analysis of APEG-GO-CA3

**Figure 6:**
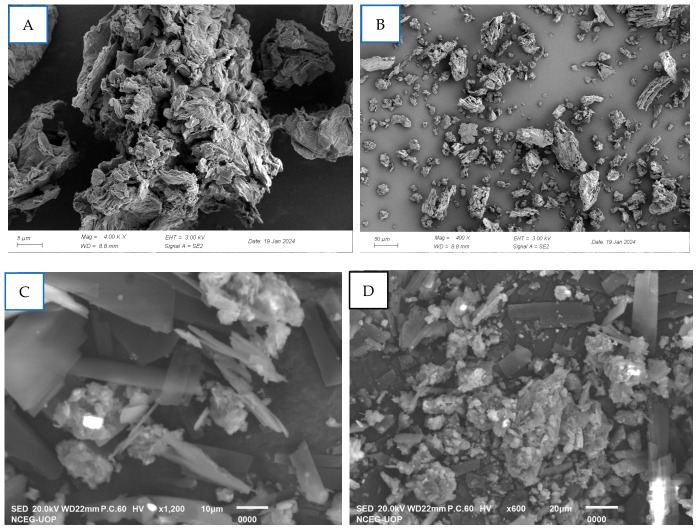
SEM images of PEG-GO (**A**,**B**); CA3-loaded PEG-GO (**C**,**D**) at different magnifications.

Scanning Electron Microscopy (SEM) was employed to examine the morphological changes in the CA loaded onto the surface of PEG-GO (Figure 6A–D). The PEG-GO micrograph shows clusters of GO sheets. The micrographs of PEGylated GO clearly indicated that PEG chains are distributed around the graphene oxide sheets, resulting in more pronounced sheet edges and a comparatively rough yet planar surface. The distinct edge morphology may also arise from hydrogen-bonding interactions or capillary forces between GO and PEG, leading to intimate mixing and compact stacking of the components. SEM images further disclose irregular fractured surfaces with visible pores, which are likely produced by partial detachment of sheet layers. This observation suggests a force-assisted incorporation of PEG within the layered GO structure (Figure 6A,B). SEM analysis at multiple magnifications shows the successful yet non uniform loading of CA onto PEGylated graphene oxide. The compound loading resulted in a change of morphology from clusters to sheets like GO. The compound is visible on the surfaces of PEGylated GO as observed in Figure 6C,D.

### 3.7. Zeta Potential and Particle Size

Zeta potential is important for providing information about the surface charge of the particle which greatly affects its interaction with cells in physiological systems, absorption in GIT and adsorption on the surface of enzymes which in turn affect the activities of enzymes. The average particle size of PEG-GO-CA3 was slightly more than 100 nm, in the acceptable range with a zeta potential of −10.2 mV (Figures S1–S3).

### 3.8. Drug-Release Study

**Figure 7:**
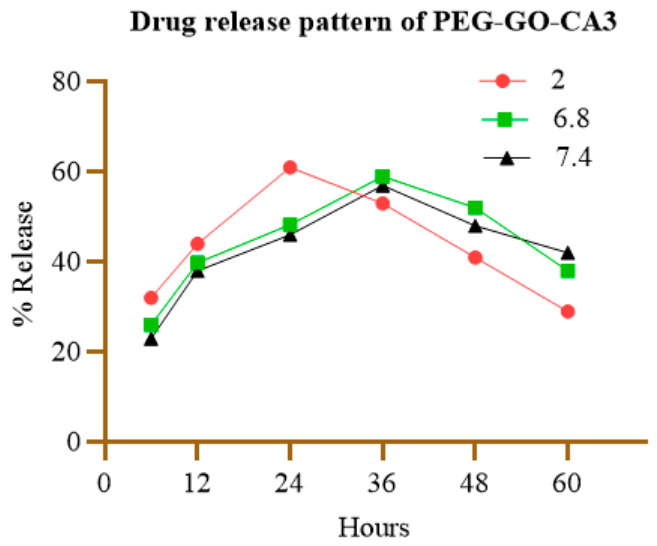
Drug-release study of PEG-GO-CA3 at pH 2, 6.8 and 7.4.

Figure 7 represents the drug-release profile. The drug-release behavior of PEG-GO was evaluated at pH 7.4, 2.0, and 6.8, representing normal physiological conditions relevant to enzyme activity and drug–body interactions, as well as simulated gastric and intestinal environments. At pH 7.4, 38% of the drug was released within the first 12 h, while the release reached 57% after 36 h. At pH 2, drug release rose sharply over the first 24 h but then declined significantly. The rate of drug release from PEG-GO gradually decreased over time, showing a sustained release pattern. At pH 6.8, the release behavior was similar to that at pH 7.4 in the initial 48 h; however, a bulk release was reported when the time exceeded 48 h.

## 4. Discussion

Curcumin and its analogues have been extensively studied for their potential in the management of inflammation, oxidative stress, diabetes, Alzheimer’s disease and cancer [40,41]. The major problem with the use of curcumin and its analogues is their poor solubility and lower bioavailability profiles, which lower their therapeutic effectiveness [42]. PEGylated graphene oxide has emerged as a promising nano carrier with a larger surface area, higher drug loading capacity, tunable size, biocompatibility and dispersibility in water. This makes PEG-GO a promising nano carrier for highly lipophilic drug delivery [21]. Curcumin has the potential to lower the blood lipid level, exert protective effects on the kidney and liver, increase insulin sensitization, promote the release of insulin and inhibit α-amylase and α-glucosidase activities. This promotes its use in the management and treatment of diabetes [43].

Control of postprandial hyperglycemia is crucial for the treatment of T2DM. Inhibiting the activities of α-amylase and α-glucosidase greatly reduces the glucose level [44]. In the current study, in vitro, in vivo and molecular docking approaches were used to study the effect of five synthesized mono-curcumin analogues for the management of T2DM. All CAs showed different potencies and affinities with α-amylase and α-glucosidase. Among the five mono-curcumin analogues, in the docking study of the compounds with a-amylase, CA3 exhibited the highest binding affinity, with a binding energy of −10.1 ± 0.3 kcal/mol, indicating strong interaction with α-amylase. CA3 formed a strong hydrogen bond with Gln63 (3.00 Å), along with hydrophobic and other bond interactions involving Trp59 (4.11 Å), His299 (4.36 Å), and Tyr62 (3.84 and 4.40 Å) of the α-amylase active site. CA3 showed a strong hydrogen bond interaction with Gly160 (3.06 Å) and hydrogen (other hydrophobic) bond interactions with Tyr158 (4.60 Å), Phe314 (4.22 Å), and Ile419 (5.47 Å) of α-glucosidase. This binding strength is followed by CA2 and CA4. The binding affinity of the compounds decreased in the order CA3 > CA2 > CA4 > CA5 > CA1. All curcumin analogues decreased postprandial hyperglycemia with some differences in potency. They decreased the percent of glycosylated Hb and improved the lipid profile, serum urea and creatinine level. CA3 loaded onto PEG-GO showed higher activity as compared to unloaded CAs. Bisdemethoxycurcumin found in *Curcuma longa* is an α-amylase inhibitor with an IC_50_ value of 63.5 mM. This compound has the potential to inhibit α-amylase function by 30%. A molecular docking study showed hydrogen bond and π-π interactions with the α-amylase enzyme, consequently being potent in reducing blood glucose level [45]. Charmi et al., 2019 synthesized graphene oxide functionalized with PEG and subsequently loaded it with curcumin. A 60% release of curcumin was observed at pH 7.4 over 96 h of time with 13.9 mV zeta potential. The study found that PEG-GO can improve the bioavailability of curcumin, thus improving its therapeutic outcomes [39].

The activity of curcumin is modified by the presence of different functional groups.

Chlorine was found to increase dipole–dipole and hydrogen-bonding interactions with the target proteins [17]. Electron-withdrawing groups produce inductive effects. These groups also increase the lipophilicity of the compounds, thereby enhancing their penetration into the helical structures of α-amylase and α-glucosidase, leading to greater inhibitory activity. Similarly, the presence of delocalized pi electrons on the methoxy curcumin analogue increases its inhibitory potential against both enzymes [46]. The study showed that the presence of a methyl group on the benzene ring decreases the compound’s activity [47]. Thakral et al., (2020) synthesized many benzamide compounds and evaluated their inhibitory potential against α-amylase and α-glucosidase. Their results suggested that the presence of electron-withdrawing groups such as chloro and nitro in curcumin derivatives showed excellent inhibitory effects. These compounds also showed higher hydrogen, hydrophobic, electrostatic and other bond interactions with α-amylase and α-glucosidase active sites, indicating greater potential in the management of diabetes [48].

In another study, many curcumin derivatives containing pyrano [2,3-d]pyrimidine heterocycles were synthesized using a multi-component reaction between curcumin, aldehydes, and barbituric acid. Among all these derivatives, the chloro derivative showed higher activity against α-amylase, while nitro-group- and 3-indolyl-group-bearing compounds showed intermediate activities. Structural modification of these curcumin analogues increased their α-glucosidase and α-amylase inhibition potential. In conclusion, the study reflected that the antioxidant potential of curcumin analogues was increased by introduction of electron-rich species into its structure [49]. Mehrabi et al., 2021 [17] synthesized thirteen curcumin derivatives (L1 to L13) containing pyrano [2,3-d]pyrimidine heterocycles in the presence of borax as a catalyst and screened them for α-amylase and α-glucosidase inhibition activities. The IC_50_ values of acarbose, L14, L12, L2 and L11 for the inhibition of α-amylase were obtained as 14.8 ± 1.5, 28.4 ± 0.3, 20.3 ± 1.3, 105 ± 2.5, and 39.3 ± 1.5 µM, respectively. IC_50_ values of acarbose, L14, L12, L2 and L11 against α-glucosidase were obtained as 14, 21, 18, 68 and 32 µM, respectively. These indicated their strong potential in lowering blood glucose levels. Molecular docking studies showed that all of these compounds made strong hydrogen bonds and hydrophobic and other bond interactions with the amino acids of α-amylase and α-glucosidase.

Halogen-group and heterocyclic substitution of curcumin increase its stability. The presence of an electron-withdrawing group increases enzyme inhibition many folds as compared to electron-donating groups [50]. In another study, a series of piperidinylamide mono-carbonyl analogues of curcumin were synthesized and screened for their biological activities against α-glucosidase/α-amylase. It was found that all of the curcumin analogues showed good α-glucosidase and α-amylase inhibitory activities. Amongst all, the compounds containing a 3,4,5-trimethoxy phenyl group, vanillin group, p-chlorophenyl group or pyrrole group showed better activities than acarbose and curcumin. Their IC_50_ values ranged from 21.2 to 55.5 µg/mL as compared to acarbose, which had a value of 34.7 ug/mL. Molecular docking studies showed their strong hydrogen bond interactions with the amino acids of α-amylase and α-glucosidase. These results of various studies confirm the potential of mono-curcumin analogues in the management of T2DM [51].

## 5. Conclusions

This study demonstrated five mono-carbonyl curcumin analogues (CA1–CA5) for their glycemic control potential, identifying CA3 as the most potent compound. CA3 exhibited strong inhibitory activity against α-amylase and α-glucosidase, which is supported by molecular docking and in vitro assays. In vivo studies further confirmed significant improvements in glycemic status, lipid profile, body weight, and hepatic and renal biomarkers. Nanoformulation of CA3 using PEGylated graphene oxide markedly enhanced its therapeutic efficacy, supporting its use in type 2 diabetes management.

## Figures and Tables

**Table 1:** The ligand molecules with an α-amylase binding score, hydrogen interaction, and hydrophobic interaction.

Compound	Binding Energy (kcal/mol)	H-Bonding Interacting Residues	Other Interactions
CA1	−7.2 ± 0.5		Lys200 (4.69 Å) Hıs201 (4.55 Å) Leu165 (3.73 Å) Ala198 (5.30 Å) Ile235 (3.38 Å)
CA2	−9.6 ± 0.6	Glu233 (2.63 Å) Gln63 (3.20 Å)	Asp197 (4.59 Å) Ala198 (5.07 Å) Leu162 (4.99 Å) Trp59 (5.52 Å)
CA3	−10.1 ± 0.3	Gln63 (3.00 Å)	Trp59 (4.11 Å) Hıs299 (4.36 Å) Tyr62 (4.40 Å, 3.84 Å)
CA4	−9.1 ± 0.5	Ala106 (3.13 Å) Asn105 (3.10 Å) Gly164 (2.37 Å) Asp300 (1.92 Å) Asp197 (2.68 Å)	Asn105 (2.54 Å)
CA5	−8.8 ± 0.4	Gln63 (3.05 Å) Asp300 (5.68 Å) Hıs299 (3.54 Å)	Tyr62 (4.98 Å, 3.73 Å) Trp59 (4.64 Å)
Control (Acarbose)	−10.6 ± 0.3	Hıs299 (2.83 Å) Asp300 (3.64 Å) Asp197 (3.56 Å) Glu233 (2.01 Å) Hıs305 (3.12 Å) Thr163 (2.74 Å) Tyr151 (3.06 Å) Ile148 (3.26 Å)	-

**Table 2:** The ligand molecules with α-glucosidase binding score, hydrogen interaction, and hydrophobic interaction.

Compound	Binding Energy (kcal/mol)	H-Bonding Interacting Residues	Other Interactions
CA1	−7.6 ± 0.6	-	Hıs280 (5.18 Å) Glu277 (4.89 Å) Asp215 (3.75 Å) Val216 (5.18 Å) Tyr72 (5.01 Å)
CA2	−9.8 ± 0.6	Arg315 (4.00 Å) Leu313 (3.19 Å) Asp215 (3.52 Å)	Arg315 (4.82 Å) Lys156 (2.25 Å) Val21 (5.34 Å) Asp352 (4.56 Å)
CA3	−10.0 ± 0.3	Gly160 (3.06 Å)	Tyr158 (4.60 Å) Phe314 (4.22 Å) Ile41 (5.47 Å)
CA4	−9.3 ± 0.6	Asp307 (2.38 Å) Glu277 (2.56 Å) Asp215 (2.38 Å)	Asp307 (3.69 Å) Arg442 (4.66 Å) Arg315 (5.10 Å)
CA5	−8.9 ± 0.4	Asp69 (3.55 Å) Asp215 (3.50 Å) Leu313 (3.64 Å) Asn415 (3.50 Å)	Hıs351 (5.40 Å) Val216 (5.33 Å) Asp352 (4.46 Å) Tyr72 (3.66 Å) Phe314 (4. 93 Å) Lys156 (4.70 Å) Arg315 (4.77 Å)
Control (Quercetin)	−9.9 ± 0.3	Asn415 (2.68 Å) Asn235 (3.67 Å) Asn317 (3.98 Å)	Hıs423 (5.25 Å) Ile419 (5.20 Å) Lys156 (4.30 Å) Phe314 (4.91 Å)

**Table 3:** In vitro IC_50_ values of α-glucosidase and α-amylase inhibition studies.

Compound	Structure	α-Glucosidase Inhibition Activity IC_50_ (µM)	α-Amylase Inhibition Activity IC_50_ (µM)
Control	Acarbose/Quercetin	21.4 ± 1.6	15.5 ± 1.7
CA1	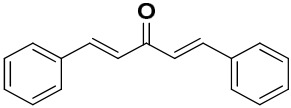	61.9 ± 2.2 ****	76.1 ± 2.4 ****
CA2	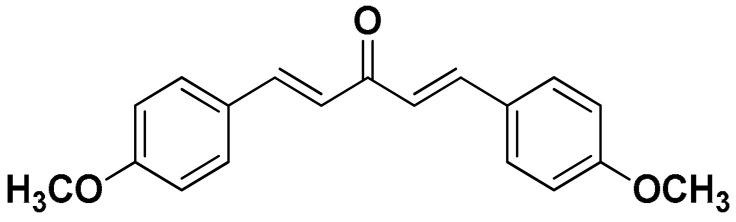	28.8 ±1.2 ****	27.1 ± 1.6 ****
CA3	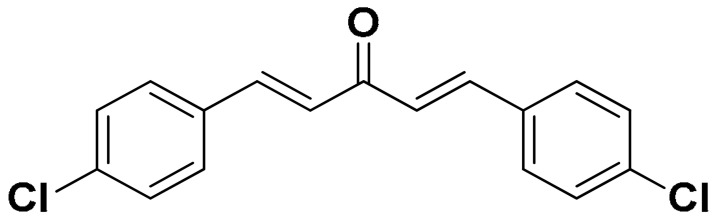	24.1 ± 2.7 ***	18.4 ± 1.8 ***
CA4	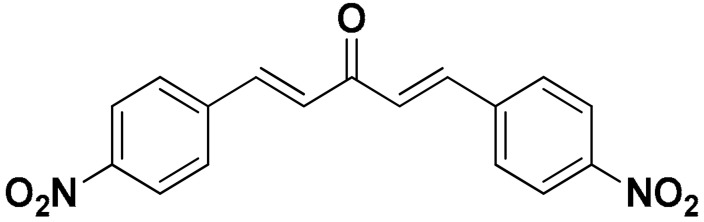	33.0 ± 1.8 ****	36.2 ± 1.9 ****
CA5	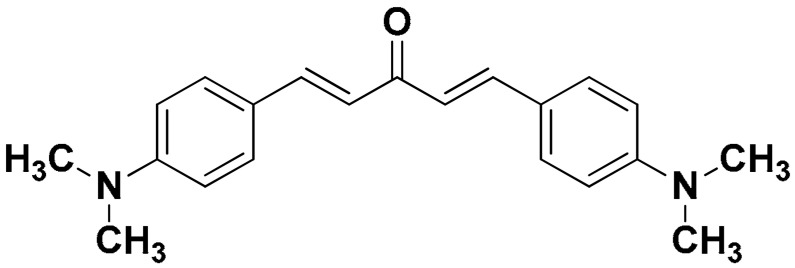	41.1 ± 1.4 ****	47.6 ± 2.6 ****
PEG-GO-CA3		20.2 ± 0.95 ^ns^	16.1 ± 1.1 ^ns^

IC_50_ value of glucosidase and amylase, **** *p* < 0.0001, *** *p* = 0.0006, ns *p* > 0.05.

**Table 4:** Effect of curcumin analogues on blood glucose levels.

Group (Dose)	Glucose Concentration (mg/dL)					
	Before Treatment	Day 1	Day 7	Day 14	Day 21	Day 28
Normal Control	119 ± 3	114 ± 3	109 ± 2	112 ± 3	116 ± 3	118 ± 2
Diabetic Control	370 ± 2	350 ± 4	372 ± 3	376 ± 4 ****	379 ± 4	384 ± 3
Acarbose (20 mg/kg)	375 ± 2	260 ± 5 ****	220 ± 2 ****	180 ± 3 ****	140 ± 3 ****	145 ± 3 ****
CA1 (15 mg/kg)	372 ± 3	359 ± 4 ^ns^	352 ± 3 ****	339 ± 3 ****	324 ± 2 ****	313 ± 3 ****
CA2 (15 mg/kg)	380 ± 3	328 ± 4 ****	309 ± 2 ****	279 ± 2 ****	262 ± 1 ****	256 ± 3 ****
CA3 (15 mg/kg)	377 ± 3	310 ± 3 ****	285 ± 2 ****	258 ± 2 ****	240 ± 2 ****	234 ± 2 ****
CA4 (15 mg/kg)	372 ± 3	341 ± 3 ^ns^	340 ± 3 ****	316 ± 2 ****	302 ± 3 ****	295 ± 1 ****
CA5 (15 mg/kg)	368 ± 5	352 ± 4 ^ns^	347 ± 4 ****	330 ± 3 ****	309 ± 2 ****	301 ± 3 ****
CA3 Loaded (15 mg/kg)	369 ± 2	295 ± 3 ****	264 ± 2 ****	223 ± 2 ****	204 ± 2 ****	191 ± 2 ****

Note: **** *p* < 0.0001, ns *p* > 0.05.

**Table 5:** Effect of curcumin analogues on the lipid profile of animals.

Group/Dose (mg/kg)	TG (mg/dL)	TC (mg/dL)	LDL (mg/dL)	HDL (mg/dL)
Normal Control	67 ± 2	54 ± 1	15 ± 1	27 ± 1
Diabetic Control	138 ± 2	74 ± 1	23 ± 1	17 ± 1
Acarbose (20 mg/kg)	86 ± 1 ****	58 ± 1 ****	16 ± 1 ****	25 ± 1 ****
CA1 (15 mg/kg)	129 ± 2 ****	70 ± 1 ***	20 ± 1 ****	18 ± 1 ^ns^
CA2 (15 mg/kg)	114 ± 2 ****	64 ± 1 ****	17 ± 0.4 ****	21 ± 1 ****
CA3 (15 mg/kg)	106 ± 2 ****	61 ± 2 ****	17 ± 1 ****	23 ± 0.2 ****
CA4 (15 mg/kg)	119 ± 2 ****	67 ± 2 ****	18 ± 1 ****	20 ± 0.1 ****
CA5 (15 mg/kg)	126 ± 1 ****	69 ± 2 ****	19 ± 1 ****	20 ± 0.1 ****
CA3 loaded (15 mg/kg)	90 ± 2 ****	57 ± 2 ****	16± 1 ****	25 ± 1 ****

Note: **** *p* < 0.0001, *** *p* = 0.0006, ns *p* > 0.05.

**Table 6:** Effect of curcumin analogues on glycosylated Hb (Hb%) of animals.

Group/Dose mg/kg	Glycosylated Hb (Hb %)
Normal control	4.4 ± 0.7
Diabetic control	10.3 ± 0.5
Acarbose (20 mg/kg)	5.3 ± 0.5 ****
CA1 (15 mg/kg)	9.6 ± 0.4 ^ns^
CA2 (15 mg/kg)	7.1 ± 0.3 ****
CA3 (15 mg/kg)	6.3 ± 0.2 ****
CA4 (15 mg/kg)	7.7 ± 0.4 ****
CA5 (15 mg/kg)	8.8 ± 0.6 ***
CA3 Loaded (15 mg/kg)	5.5 ± 0.2 ****

Note: **** *p* < 0.0001, *** *p* = 0.0006, ns *p* > 0.05.

**Table 7:** Effect of curcumin analogues on urea, creatinine, ALT, and AST in mice.

Group/Dose (mg/kg)	Urea (mg/dL)	Creatinine (mg/dL)	ALT (U/L)	AST (U/L)
Normal control	35 ± 2	0.86 ± 0.08	67 ± 2.4	52.4 ± 1
Diabetic control	103 ± 3	2.1 ± 0.03	126 ± 3	103 ± 2
Acarbose (20 mg/kg)	41 ± 2 ****	1.2 ± 0.02 ****	73 ± 2.5 ****	64 ± 2 ****
CA1 (15 mg/kg)	79. ± 2 ****	1.8 ± 0.05 ****	109 ± 1 ****	93 ± 2 ****
CA2 (15 mg/kg)	60 ± 2 ****	1.6 ± 0.09 ****	94 ± 3 ****	83 ± 2 ****
CA3 (15 mg/kg)	56 ± 3 ****	1.5 ± 0.04 ****	86 ± 2 ****	76 ± 2 ****
CA4 (15 mg/kg)	62 ± 3 ****	1.6 ± 0.05 ****	96 ± 3 ****	85 ± 1 ****
CA5 (15 mg/kg)	78 ± 3 ****	1.7 ± 0.02 ****	112 ± 3 ****	90 ± 2 ****
CA3 loaded (15 mg/kg)	48 ± 2 ***	1.2 ± 0.02 ****	71 ± 1 ****	62 ± 2 ****

Note: **** *p* < 0.0001, *** *p* = 0.0006, ns *p* > 0.05.

**Table 8:** Effect of curcumin analogues on body weight of animals.

Group and Dose mg/kg	Day 1	Day 7	Day 14	Day 21	Day 28
Normal control	212 ± 4	218 ± 3	223 ± 3	228 ± 3	233 ± 3
Diabetic control	214 ± 3	206 ± 4	194 ± 3	183 ± 3	171 ± 2
Acarbose (20 mg/kg)	209 ± 3 ^ns^	208 ± 3 ^ns^	205 ± 3 **	203 ± 2 ****	201 ± 3 ****
CA 1 (15 mg/kg)	210 ± 3 ^ns^	203 ± 4 ^ns^	197 ± 3 ^ns^	190 ± 4 ^ns^	179 ± 2 *
CA 2 (15 mg/kg)	212 ± 2 ^ns^	207 ± 4 ^ns^	203 ± 3 *	198 ± 2 ****	189 ± 4 ****
CA 3 (15 mg/kg)	215 ± 3 ^ns^	209 ± 2 ^ns^	204 ± 2 **	202 ± 2 ****	192 ± 3 ****
CA4 (15 mg/kg)	214 ± 3 ^ns^	208 ± 4 ^ns^	203 ± 3 *	197 ± 3 ***	191 ± 4 ****
CA5 (15 mg/kg)	212 ± 4 ^ns^	206 ± 3 ^ns^	199 ± 4 ^ns^	191 ± 3 *	182 ± 2 **
CA3 loaded (15 mg/kg)	215 ± 1 ^ns^	211 ± 3 ****	208 ± 2 ****	205 ± 2 ****	201 ± 2 ****

Note: **** *p* < 0.0001, *** *p* = 0.0006, ** *p* = 0.005, * *p* = 0.01, ns *p* > 0.05.

## Data Availability

Data is available from the corresponding author on request.

## Supplementary Materials

The following supporting information can be downloaded at: https://www.mdpi.com/article/10.3390/pharmaceutics18050568/s1, Figure S1: (A) Zeta potential; (B) Particle size measurements of GO; Figure S2: Zeta potential; Figure S3: Particle size measurements of GO.

## Abbreviations

The following abbreviations are used in this manuscript:

AST	Aspartate transaminase
ALP	Alkaline phosphatase
DM	Diabetes mellitus
GO	Graphene oxide
HDL	High-density lipoprotein
LDL	Low-density lipoprotein
NIDDM	Non-insulin-dependent diabetes mellitus
PEG	Polyethylene glycol
STZ	Streptozotocin
TC	Total cholesterol
TGs	Triglycerides
