# Supplementary material for: Nano-Enabled Potentiation of a Lead Mono-Carbonyl Curcumin Analogue via PEGylated Graphene Oxide for Enhanced Glycemic Control
Source: Pharmaceutics. 2026 May 2;18(5):568. doi: 10.3390/pharmaceutics18050568 (PMC13210871; doi:10.3390/pharmaceutics18050568)
Supplement: Supplementary file 1 [file pharmaceutics-18-00568-s001.zip › pharmaceutics-4233025-supplementary.pdf]

Supplementary material

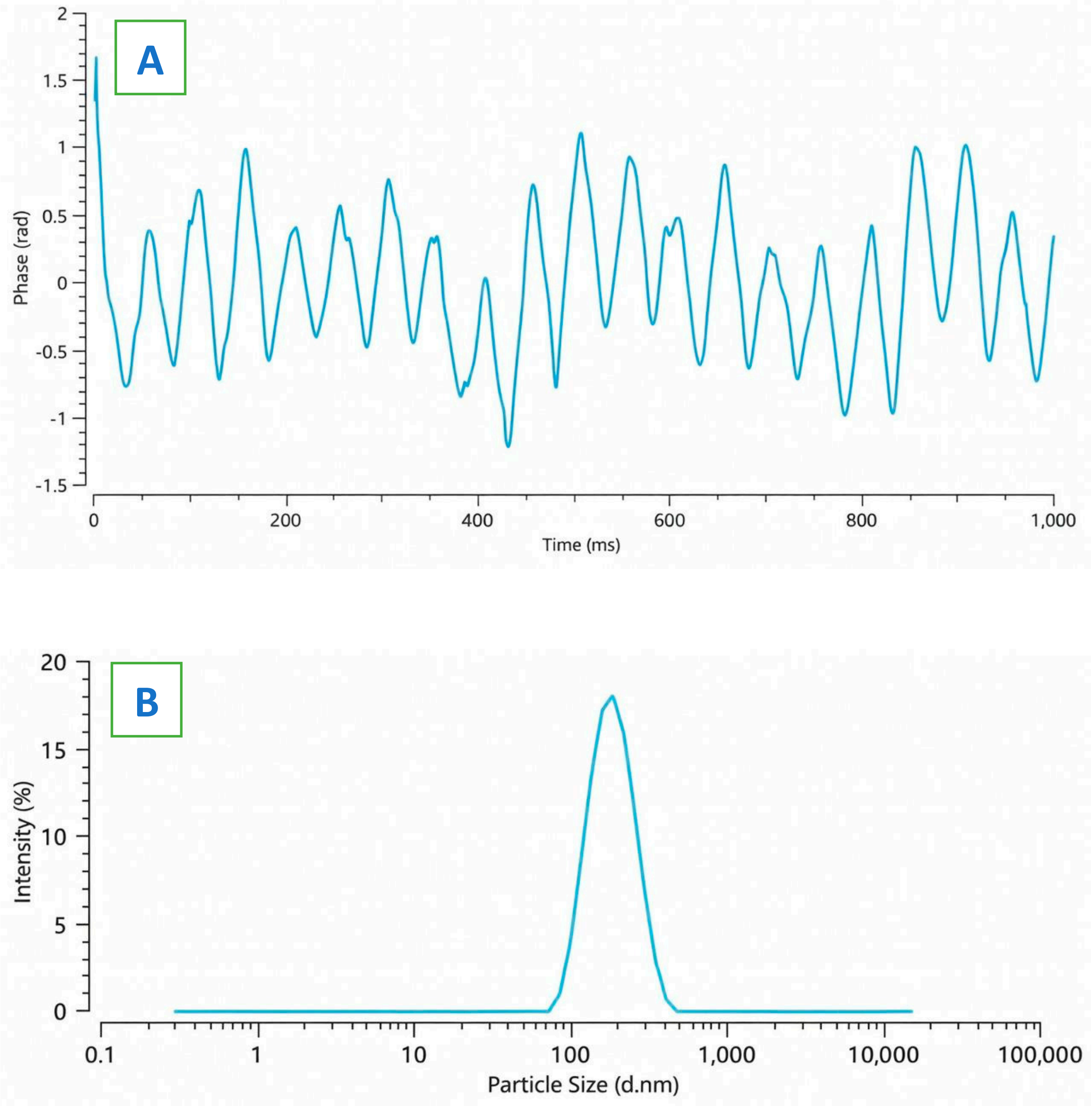

Figure S1: (A) Zeta potential; (B) Particle size measurements of GO.

百特纳米粒度电位分析仪

Zeta电位均值报告页 V4.20

样品名称：H2O-Et-Day 2-2	样品来源：
检测人员：FWD	检测单位：
SOP名称：manual.sop	测试时间：2026/1/19 10:11:59
备注：	

介质名称：水	波长：671 nm	子测试次数：12
温度：25°C	有效电压：150.04 V	衰减片编号：4
介质粘度：0.8936 mPa.s(cP)	分析模式：快场	样品池种类：毛细管电极
介质折射率：1.33	平衡时间：120 s	pH值：

测试结果：

Zeta电位：-10.2115 mV

电泳迁移率：-0.7950 μm.cm/V.s

电导率：0.0513 mS/cm

	平均Zeta电位 (mV)	面积 (%)	峰标准差 (mV)
峰1			
峰2			
峰3			

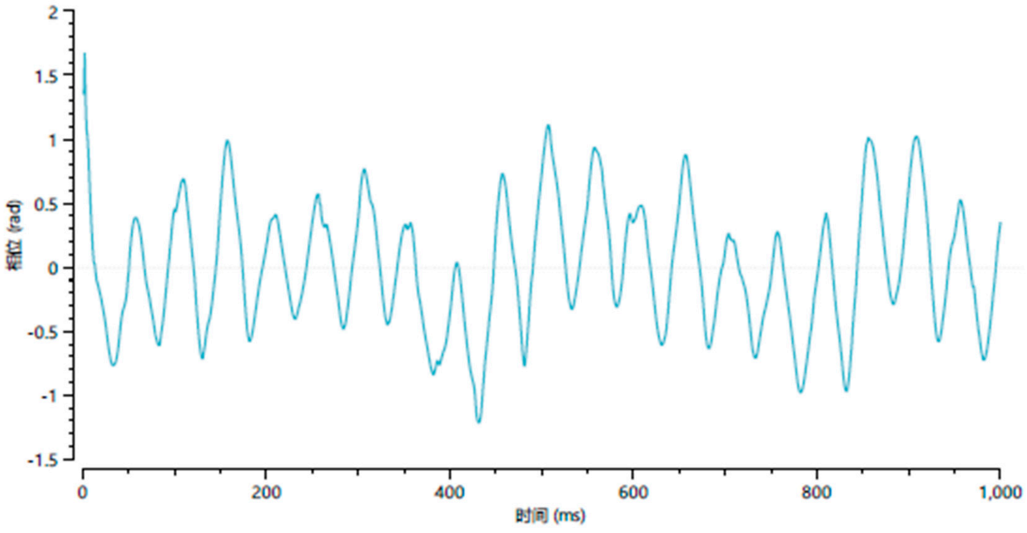

Figure S2: Zeta potential

百特纳米粒度电位分析仪

光强分布报告页　　V4.20

样品名称：H2O-Et-Day 2-1	样品来源：
检测人员：FWD	检测单位：
SOP名称：manual.sop	测试时间：2026/1/19 10:21:13
备注：	

介质名称：水	散射角度：173 °	子测试次数：10
温度：25 °C	平均光强：999.37 kcps	衰减片编号：5
介质粘度：0.8936 mPa.s(cP)	分析模型：通用模式	样品池类型：PS样品池
介质折射率：1.33	平衡时间：120 s	检测点位置：3.9 mm

测试结果：

Z-均粒径：185.08 nm

PDI：0.022

截距：0.90

	均径 (d.nm)	面积 (%)	峰标准差 (nm)	CV (%)
峰1	191.01	100.00	62.30	32.62
峰2				
峰3				

粒径 (d.nm)	区间 (%)	累积 (%)	粒径 (d.nm)	区间 (%)	累积 (%)	粒径 (d.nm)	区间 (%)	累积 (%)	粒径 (d.nm)	区间 (%)	累积 (%)
0.30	0.00	0.00	5.05	0.00	0.00	84.87	0.97	0.97	1427.50	0.00	100.00
0.35	0.00	0.00	5.90	0.00	0.00	99.28	3.84	4.81	1669.85	0.00	100.00
0.41	0.00	0.00	6.90	0.00	0.00	116.13	8.48	13.29	1953.34	0.00	100.00
0.48	0.00	0.00	8.08	0.00	0.00	135.85	13.53	26.82	2284.96	0.00	100.00
0.56	0.00	0.00	9.45	0.00	0.00	158.91	17.19	44.02	2672.88	0.00	100.00
0.66	0.00	0.00	11.05	0.00	0.00	185.89	18.08	62.10	3126.66	0.00	100.00
0.77	0.00	0.00	12.93	0.00	0.00	217.45	15.93	78.03	3657.48	0.00	100.00
0.90	0.00	0.00	15.12	0.00	0.00	254.37	11.63	89.65	4278.42	0.00	100.00
1.05	0.00	0.00	17.69	0.00	0.00	297.55	6.78	96.43	5004.77	0.00	100.00
1.23	0.00	0.00	20.69	0.00	0.00	348.07	2.88	99.31	5854.44	0.00	100.00
1.44	0.00	0.00	24.21	0.00	0.00	407.16	0.69	100.00	6848.35	0.00	100.00
1.68	0.00	0.00	28.32	0.00	0.00	476.29	0.00	100.00	8011.01	0.00	100.00
1.97	0.00	0.00	33.12	0.00	0.00	557.15	0.00	100.00	9371.05	0.00	100.00
2.30	0.00	0.00	38.75	0.00	0.00	651.73	0.00	100.00	10961.99	0.00	100.00
2.69	0.00	0.00	45.33	0.00	0.00	762.38	0.00	100.00	12823.02	0.00	100.00
3.15	0.00	0.00	53.02	0.00	0.00	891.81	0.00	100.00	15000.00	0.00	100.00
3.69	0.00	0.00	62.02	0.00	0.00	1043.22	0.00	100.00			
4.31	0.00	0.00	72.55	0.00	0.00	1220.32	0.00	100.00			

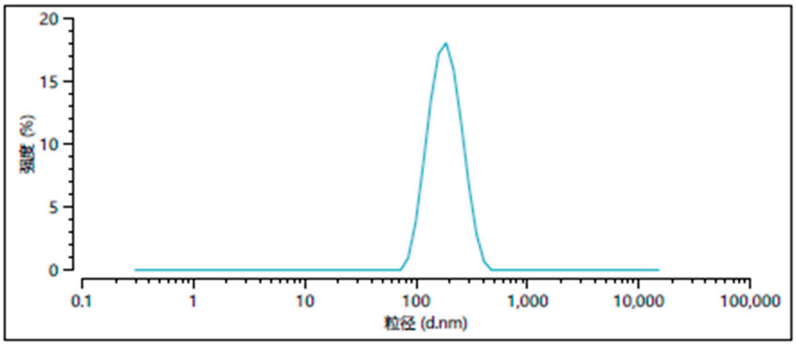

典型值	粒径 (d.nm)
D10	109.59
D20	125.91
D30	140.11
D40	153.53
D50	167.84
D60	182.76
D70	201.55
D80	223.72
D90	256.57
D100	407.16

仪器制造商：丹东百特仪器有限公司 网址：www.bettersize.com 邮箱：bettersize@sohu.com 电话：0415-6184440

2026-01-19 10:23:18

Figure S3: Particle size measurements of GO.
